# Reduced Cochlear Implant Performance in Listeners with Single-Sided Deafness: Comparison with Bilateral Listeners

**DOI:** 10.1007/s10162-025-01001-3

**Published:** 2025-07-14

**Authors:** Charlotte Jeppsen, Bob McMurray

**Affiliations:** 1 Department of Psychological and Brain Sciences, University of Iowa, Iowa City, IA USA; 2 Department of Communication Sciences and Disorders, University of Iowa, Iowa City, IA USA; 3 Department of Languages, Linguistics, Literatures and Cultures, University of Iowa, Iowa City, IA USA; 4 Department of Otolaryngology, University of Iowa, Iowa City, IA USA

**Keywords:** Single-sided deafness, Speech perception, Bilateral cochlear implants, Audiology

## Abstract

**Purpose:**

The efficacy of the Cochlear Implant (CI) in listeners with single-sided deafness (SSD) was evaluated by comparing single-ear speech perception in SSD listeners and bilateral cochlear implant listeners (BCI).

**Methods:**

Consonant-nucleus-consonant (CNC) speech perception scores for the CI-only ear in SSD listeners (N = 55; 36 female, 19 male) were compared to single-ear performance in age and device experience-matched BCI listeners (N = 55; 29 female, 26 male). Separate analyses examined: (1) a matched ear from the BCI listeners (for sequentially implanted BCI listeners, the first-implanted ear in sequential BCI listeners, or, for simultaneously implanted BCI listeners, the ear on the same side as the CI in the matching SSD listener), and (2) the lower-performing ear across BCI listeners. Additional models included moderators such as age, time since activation, CI usage, and etiology. A final analysis compared first and second implants for sequential BCI listeners.

**Results:**

SSD listeners showed significantly lower CNC performance after controlling for age, time since activation, CI usage, and etiology. Sequential BCI listeners exhibited significantly lower CNC performance on their second ear, compared to their first ear.

**Conclusion:**

Speech perception with CIs is reduced in SSD listeners compared to BCI users, likely due to blocking, where the normal-hearing ear diminishes reliance on the CI. Lower performance in the second implanted ear of sequential BCI listeners also suggests greater reliance on the more experienced ear. These findings highlight the need for additional training, resources, and support to optimize CI performance in SSD listeners, despite prior evidence of positive CNC outcomes.

## Introduction

Single-sided deafness (SSD) occurs when a listener has near-normal hearing in one ear and severe-to-profound hearing loss in the other ear (pure-tone average threshold ≥ 70 dB HL) [1]. SSD can derive from a variety of factors, including birth defects, trauma to the ear, and diseases or infections that affect the ear [2]. SSD listeners typically rely on their normal hearing (NH) ear, as one ear may be sufficient for many listening situations (e.g., speech perception in quiet, music enjoyment) [3–5]. However, increasing numbers of SSD listeners receive a cochlear implant (CI) on their deafened side. The disparity between the input afforded by the NH ear and the CI raises important scientific and clinical questions about the benefits of this listening configuration, and about how people integrate disparate inputs from electric and acoustic hearing.

One reason for CI use in SSD listeners is that the addition of the second ear (via the CI) could enable better spatial release from masking, in which listeners use the location of an attended target as a cue to segregate it from noise coming from another source. This depends on the ability to localize sounds, where binaural hearing is advantageous. NH listeners typically localize sounds using Interaural Time and Level Differences (ITDs, ILDs), (see [6] for a review). However, spatial release from masking can also be achieved via more basic processes like head shadow (a sound is perceived to be quieter in the ear not facing the noise) [7], or squelch (selective attention to the ear with the signal) [8]. Regardless of the mechanism, having two ears to help with these processes is highly beneficial to listeners when perceiving speech in noisy situations where the noise is not co-localized with the speech [9, 10]. For instance, prior work shows that the CI enhances directional hearing and speech perception in noise for SSD listeners, which has been attributed to benefits of sound source localization and spatial release [11–13].

The CI may also offer additional benefits for SSD listeners that do not derive from localization. In principle, the availability of two ears may help to identify speech content. A CI could be helpful for detecting and discriminating sounds on the side of the deaf ear (given head shadow). It may also help perceive sounds that are centrally localized as it affords opportunities to encode the input, which leads to a more comprehensive percept of the sound [14, 15]. For example, this redundancy gain could help listeners perceive speech in quiet, where binaural presentation is thought to provide about a 2–3 dB benefit [16].

It is difficult to know if SSD listeners with a CI can achieve these benefits for content integration. Most of the studies documenting a benefit in speech perception for this group has tested speech in a noisy environment in which the speech comes from a different location than the noise [17–23]. In doing this, any benefits in speech perception from the CI could derive from spatial release from masking, as opposed to binaural integration of content. One study, however, tested SSD listeners with co-localized speech and noise [20]. Interestingly, this study found that SSD listeners performed worse when they listened with both ears, relative to when they only listened through their NH ear. This suggests that the CI may negatively impact speech perception in SSD listeners. This could be the case if listeners struggle to combine percepts from each ear that may be radically different (a spectrally degraded electrical pulse train vs. typical acoustic hearing). However, this study did not isolate speech perception in the CI ear alone, making it difficult to know the source of the negative performance.

Another study explicitly compared performance between isolated CI, acoustic hearing, and bilateral listening in a small group of SSD listeners who were tested on speech perception in the presence of a competing talker [24]. In the first experiment, the target speech was presented only to the CI (with either silence or competing speech in the NH ear). They found significantly reduced speech perception accuracy in the CI when competing speech was simultaneously presented in the NH ear compared to silence in the NH ear. This suggests that speech perception in the NH ear may interfere with CI speech perception. The conditions in the second experiment were reversed. The target speech was presented to the NH ear, with either silence or competing speech presented in the CI ear. This experiment found an improvement in speech perception accuracy in the NH ear when competing speech was simultaneously present in the CI ear, suggesting the CI can help unmask the NH ear. The authors of this study claim that it was more difficult for listeners to ignore salient acoustic speech in the NH ear when focusing on CI speech perception, leading to the divergent results between experiments. These results provide evidence that SSD CI users may rely upon the NH ear during speech perception, possibly to the decrement of CI performance (even as the CI can provide a benefit in some cases).

Research with bimodal CI users, in which listeners combine a traditional CI with a hearing aid, offers some additional, converging insight into the relationship between asymmetric acoustic and electric hearing. As there is usually some hearing loss in the NH ear, the main research question focuses on whether some NH offers a benefit over and above the CI alone, rather than whether the CI offers a benefit *over and above the NH ear*. This work suggests that NH is generally helpful for speech perception [25]. However, in some cases, difficulty with integrating two inputs from different modalities (electric and acoustic) yields little benefit [15, 26–28]. For instance, when categorizing vowels in binaural and monoaural conditions, many bimodal listeners show a pattern of ear dominance, ignoring their NH ear to solely rely on the CI [28]. This suggests that there may be challenges for integrating acoustic and electric hearing (supporting a potential cost for adding a CI to an NH ear). It is much less clear how the CI interacts with an unaffected NH ear in SSD listeners.

A critical missing link in understanding these issues is to determine how well the CI encodes information on its own in SSD listeners. If the CI does not encode the auditory input accurately, it may not provide much benefit over and above the NH ear alone. Or, CI speech perception may be even more difficult due to the presence of an NH ear compared to CI speech perception in fully deaf listeners. So why might CI encoding differ in SSD CI listeners? All CI listeners require an adaptation period of at least six months, and possibly up to a year [29–31]. Listeners need to adjust the mapping between location on the cochlea and particular frequencies (place pitch mapping); they may need to learn to separate frequencies; and for speech, listeners must remap the degraded auditory input (e.g., less frequency separation) to the phonological categories and lexical templates they already have.

This adaptation process may occur differently in the presence of an NH ear. Broadly speaking, there are two possibilities for the nature of this adaptation. Both start from the fact that the CI input is perceptually ambiguous (or less familiar) than the listener’s typical expectations. The first possibility is a form of *bootstrapping* in which the NH ear gives an unambiguous encoding of the speech input which can serve as a teaching signal to train the auditory system to adapt to the new input. Bootstrapping is often observed in studies of how listeners adapt to vocoded speech [32–34]. In these studies, the presence of the written form of the speech alongside the vocoded input offers a boost to training. Some work has expanded to benefits of written training on learning a second language (L2) [35]. (It is notable that this has not been attempted with unvocoded speech in L1 as the teaching stimulus as would be the case for this model of SSD adaptation). Thus, although bootstrapping is well attested in well-defined laboratory learning paradigms, it is unclear whether such a mechanism would generalize to the much more unconstrained adaptation that must occur for CI listeners post-implantation.

An alternative possibility is a form of *blocking.* Here, because the NH ear gives a near perfect encoding of the auditory input, the listener has less need to adapt to the CI. In fact, at least during early adaptation, the CI may provide significant distraction from the NH ear or may disrupt its perceptual encoding. This may encourage listeners to attend to it even less (to squelch it). Simply put, there is less of a need to adapt to the CI (than there would be for a fully deaf listener). Supporting this, some work shows that both temporal and spectral compression impede binaural fusion in NH listeners [36, 37]. This suggests that the unilateral spectral and temporal compression that is created from the CI may prohibit SSD CI listeners from binaural fusion between two ears. A lack of binaural fusion then leads to worse ultimate performance. This is consistent with basic learning principles from classical conditioning (e.g. [38],).

These two hypotheses differ on whether adaptation to the CI (operationalized as performance with the CI alone) is better or worse than would normally be expected for a fully deaf individual (e.g., how well someone would adapt without the bootstrapping or blocking afforded by the NH ear) receiving a CI for the first time (with no supervisory or blocking stimulus from the other ear).

A few recent studies have addressed this by comparing a group of recently implanted SSD listening through their CI alone to unilateral CI users who would not have had access to acoustic hearing during the adaptation period [39, 40]. Both studies found that in their CI ear alone, SSD patients had CNC (word recognition in quiet) scores that were on average 11.3% and 8% lower than unilateral, bilaterally deaf CI users. Similarly, AZBio (sentence recognition in noise) scores were on average 14.4% and 12% lower than unilateral, bilaterally deaf CI listeners. These group differences would appear to support a *blocking *account for SSD listeners.

However, there are two reasons to revisit this question. First, the small samples of 33 and 12 SSD listeners in these studies only had around 1–3 years of experience with their CI. Learning and adaptation are likely to shape CI performance in this group. However, the longitudinal course of adapting to the CI is less well characterized in SSD listeners than in profoundly deaf listeners. It is possible that the altered adaptation environment for SSD listeners could lead to slower adaptation (e.g., it takes longer to see the benefit of bootstrapping), because they must overcome conflicting inputs from the NH ear and the electric ear. Thus, it would be useful to examine more experienced CI users where we can be confident these processes have reached asymptote.

Second, unilateral CI users may not be the appropriate comparison group for SSD listeners. Forcing an SSD listener to only use their CI may result in a performance decrement, not because the CI is worse, but because they are listening in an unfamiliar configuration (one CI only). Thus, unilateral CI listeners, who always listen with a single CI, may not be a good comparison. In contrast, bilateral CI (BCI) listeners may offer a better comparison. BCI listeners have simultaneous deafness to both ears, but they can be tested in one ear (which is also an unfamiliar listening situation).

Thus, the current study sought to investigate speech perception ability in experienced SSD versus BCI listeners using a simple speech perception task in a larger sample with substantial device experience.

## Methods and Materials

### Materials

This was a retrospective analysis of a set of data collected by a larger NIH funded project at the University of Iowa. The study captured the speech perception performance of SSD and BCI participants using the Consonant-Nucleus-Consonant (CNC) word recognition test [41]. This metric was chosen over richer tests that were conducted (e.g., sentences in noise) as it offers a more pure encoding of the degree to which the peripheral auditory system (including the CI) discriminates speech-relevant cues, with fewer demands on working memory, sentence processing, etc.

### Participants

Participants were tested during their yearly audiological check-up at the UIHC Speech and Hearing Clinic. Testing was conducted after device tuning to reach the manufacturers’ specified detection thresholds (using Electrical Evoked Stapedial Reflex Thresholds, eSRTs), to ensure that all listeners were achieving sound detection with their CI within the upper stimulation level.

For most listeners in this study, CNC word recognition was assessed three times: once for each ear, and once in the listeners’ everyday listening configuration (e.g., both CIs, a CI + a NH ear, etc.). For NH and combined listening, words were presented via a single loudspeaker in front of the subject at 60 dB HL in a soundproof booth. For SSD listeners in CI only testing, testing was done through a direct connection via Bluetooth to their implant to avoid interference from the NH ear.

Responses were scored by a trained audiologist for both phonemes and words said correctly, the latter of which was used here. CNC recognition scores are saved in a research registry spanning about 30 years of CI research.

The larger registry included listeners with a variety of listening configurations (unilateral and BCI listeners, SSD CI listeners, pre- and post-lingually deaf individuals, etc.). From this dataset, SSD participants who met the following criteria were identified: (1) post lingual deafness, defined as receiving a CI after the age of 15 years, and (2), no less than 18 months had elapsed since the implantation surgery. SSD participants for this research study were then identified as having an unaided PTA in their better hearing ear as < = 45 dB HL. This is slightly higher than the typical 40 dB cutoff of the NH ear in SSD users as defined by [1] for receiving intervention. However, participants in this study were included with a PTA of 45 in their NH ear to expand the SSD sample by a few participants.

Once the list of SSD participants was identified, each SSD listener was matched to a BCI participant on chronological age, with a difference of no more than 5 years. This resulted in a final sample of 55 SSD listeners (Mean age = 62.3, SD = 9.3) and 56 BCI listeners (Mean age = 60.9, SD = 10.7). SSD participants were implanted with devices from all major CI manufacturers, while BCI participants were implanted with either Cochlear or Advanced Bionics CIs. Out of concern for protecting identifying information from our participants, a select subset of identifying information per participant is included on the Open Science Framework https://osf.io/vs6g3/. Other demographic characteristics of the groups are listed in Table 1.

**Table 1:** Demographic characteristics of the sample

Characteristic	BCI (*N* = 55)	SSD (*N* = 55)
Biological sex		
Female	36 (65%)	29 (53%)
Male	19 (35%)	26(47%)
Race		
White	54 (98%)	53 (96%)
Black or African American	0 (0%)	1 (1.8%)
American Indian/Alaskan Native	1 (1.8%)	0 (0.0%)
Other	0 (0.0%)	1 (1.8%)
Ethnicity		
Hispanic	0 (0%)	4 (7.3%)
Non-Hispanic	55 (100%)	51 (92.7%)
Device manufacturer		
Cochlear	34 (61.8%)	34 (61.8%)
Advanced Bionics	21 (38.2%)	10 (18.2%)
Med-El	0 (0%)	11 (20%)

### Data Analysis

Our primary analysis compared CNC performance between BCI and SSD listeners. There was some uncertainty about which ear to choose for the BCI listeners. Thus, the analysis was conducted in two ways. First, each analysis initially compared SSD performance to the CNC scores from the *best matched ear* in BCI listeners. For sequentially implanted BCI participants (N = 32), this was the *first implanted ear* in (since the SSD listeners would also be tested on their first implanted ear). For simultaneously implanted BCI listeners (N = 22), this was the ear on the same side (left or right) as was implanted in SSD listeners. Second, for a more conservative analysis, the same comparisons were made using the BCI users’ worse performing ear. This ensured that the results were not influenced by idiosyncrasies of how the ears were matched.

As it was not possible to match the groups on all possible factors that are known to impact speech perception, this raises the possibility that some uncontrolled factor differs among the groups could be responsible for any group differences that could be observed. To account for this, several potential moderators and mediators on group differences in CNC scores were considered.

#### Age

Even as participants were broadly matched for age, it was possible that effects differed by age, as word recognition accuracy is affected by domain general cognitive abilities, such as inhibition [42], which declines with age [43]. Therefore, age was included as a continuous moderator on group.

#### Time Since Activation

Although listeners were not matched on this factor, all listeners had at least 18 months with their CI, at which point they were expected to be fully adapted to their implant [31, 44]. Nonetheless, SSD could be exhibiting lower CNC scores if they are less experienced with their CI or if they took longer to adapt to their CI than traditional CI users. Therefore, it could be possible that group differences in CNC performance are due to difference in CI experience. To answer this, a *t*-test was initially run looking at whether there were group differences in time since activation. Following this, time since activation was included as a moderator of group differences.

#### Etiology of Deafness

It is possible that etiology of deafness could lead to variability in speech perception. That is, the likely causes of single-ear deafness (trauma, tumors) may differ markedly from bilateral deafness (SNHL), and these sources of deafness may lead to differences in the physiology of the cochlea that are relevant for the ability of the CI to activate the auditory nerve (e.g., the electro-neural interface).

Thus, etiology of deafness was examined as a moderator. Etiology was available for 31 Bilateral and 44 SSD participants. Table 2 shows a rough breakdown of the various etiologies in each group, suggesting a similar distribution of etiologies. To control for the wide variety, etiology was modeled as a factor variable with 3 levels (1 = SNHL, 2 = Meniere’s, 3 = Other; listeners with unknown etiologies were excluded from this analysis).

**Table 2:** Number of subjects with each etiology of deafness as a function of group

Etiology	Bilateral	SSD
None listed	24	10
SNHL	14	22
Meniere′s	6	15
Unknown	2	2
Noise induced	1	1
Autoimmune	1	1
Car accident	1	0
Genetic mutation	1	0
Hepatitis	1	0
Head trauma	0	1
Hereditary	1	0
Infection	1	0
Left temporal bone fracture	0	1
Usher syndrome	1	0
Viral encephalitis	1	0
Viral labyrinthies	0	1
Viral polyneuropathy	0	1

#### Device Usage (Hours per Day)

Finally, time since activation is not sufficient to capture *experience* with the device, as different types of listeners may use their CI in variable ways. Although literature suggests that SSD listeners do not differ in usage compared to BCI listeners [45, 46], a common clinical observation is that SSD listeners use their CI less, likely because their NH ear is sufficient for many purposes. Luckily, many CIs now log hours of usage, allowing for a more direct estimate. Therefore, the average hours of CI use each day was added as a moderator. This data logging feature was only available for 18 participants per group. Therefore, only these 36 subjects were examined. An initial *t*-test was conducted to determine whether there were any differences in device usage (in hours per day) between groups. Device usage was also included as a moderator.

#### Device Manufacturer

Because CI manufacturer may influence performance, separate models examined whether device manufacturer moderated group differences in CNC performance within the matched groups. This was done in a model with a contrast that examined whether Advanced Bionics (0.5) differed from Cochlear (− 0.5). Subjects with Med-El devices were excluded from this analysis, as only SSD listeners used Med-El devices in this sample. Therefore, a separate ANOVA compared whether there was an effect of device manufacturer within the SSD group, including Med-El, using a categorical factor variable with 3 levels (AB, Cochlear, Med-El).

#### Analysis Plan

Given our interest in these moderators, rather than using simple *t*-tests to compare groups, analyses were conducted in linear regression framework. Group was modeled as a single dichotomous predictor with BCI listeners as the reference group. CNC score was the dependent variable. With no other factors in the model, this is formally equivalent to a *t*-test. However, the regression framework permits us to add moderators and mediators as additional factors in the same analytic framework.

Given our large sample size (of 110 listeners), and the fact that not all moderators were available for all individuals, it was not prudent to construct one large model with all the factors, as this could have overfit the data. Additionally, collinearity among some predictors could lead to suppression. Thus, to examine these, separate models were run for each moderator. In these models, both a main effect of the moderator (centered) and its interaction were added with group. The more complex (full) model including the moderator was initially compared to the baseline model (only including group) to look for the presence of an effect. The significance of the SSD vs. BCI contrast was investigated when the additional factor was in the model (analogous to a direct effect in a mediation model). If (1) the addition of the moderator and its interaction did not significantly improve model fit; and (2) the main effect of group was still significant in the presence of the moderator, this suggests the moderating factor was not playing a role. However, if either criterion were met, the full results were then interpreted to unpack the nature of the relationships. Due to the missing data for both device usage and etiology, initial group models were run separately on the relative subsets of subjects who had these data.

All data analytic scripts are made publicly available on the Open Science Framework at https://osf.io/vs6g3/.

## Results

### Main Group Difference

**Fig. 1:**
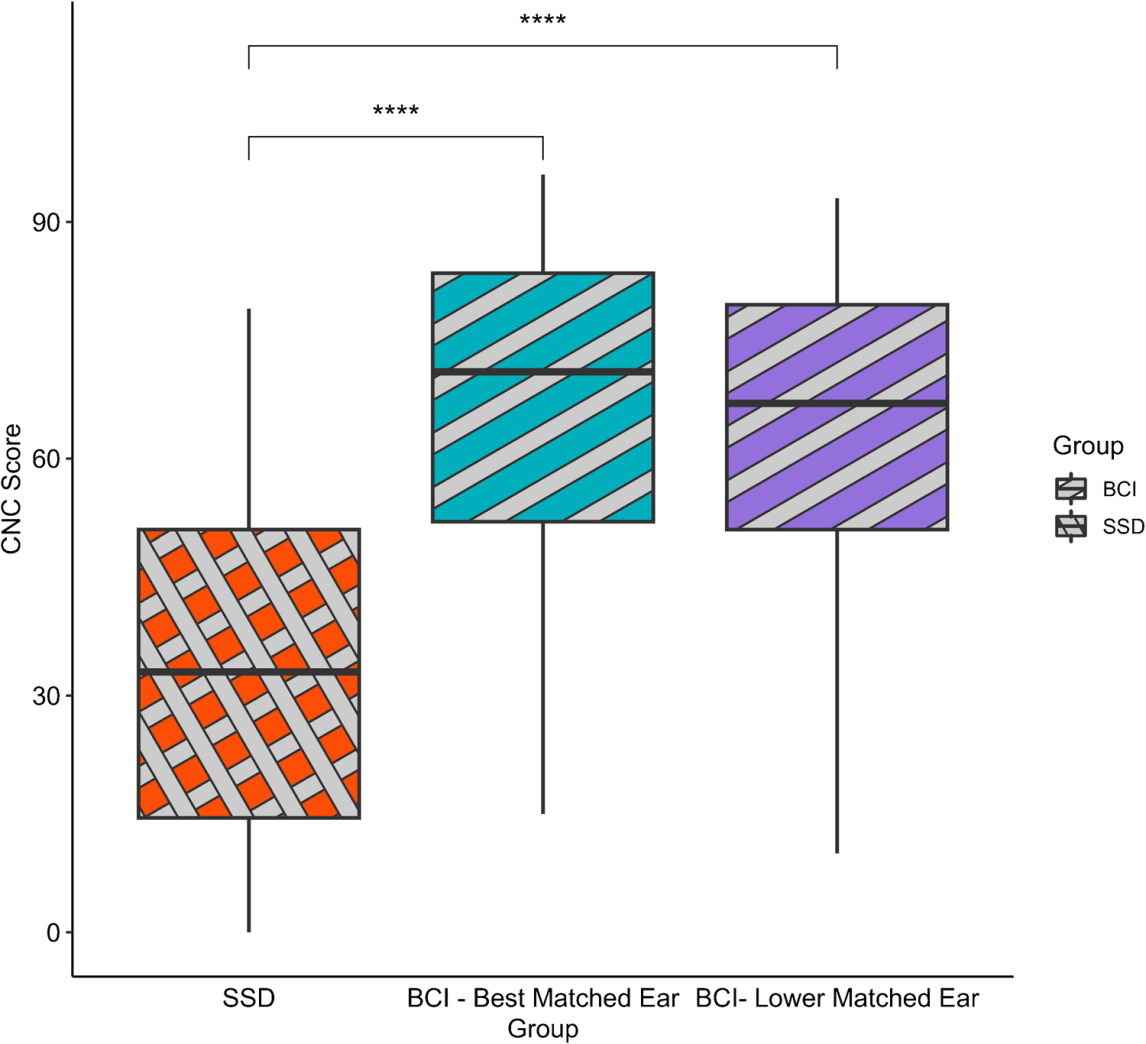
CNC performance by CI group and matching condition

Figure 1 and Table 3 show group performance on CNC scores across comparison types (which BCI ear was used). Across comparison types, SSD listeners performed significantly worse than BCI participants on the CNC task (Rows 1–6). This difference was significant regardless of whether the analysis was performed on the best matched ear (Row 1) or worse matched ear (Row 2) for BCI listeners. In preparation for the moderation analyses examining the effect of device use or etiology of deafness, group differences for just the subjects that had device usage data and etiology data were examined to ensure the effects held in these subsets of the data. Again, this was done separately for both matching conditions. These analyses found similar differences between groups compared to the full sample, with BCI listeners scoring between 20 and 40% higher than SSD listeners (Rows 1–6).

**Table 3:** Model results for group differences in CNC scores. BCI listeners represented the intercept

Row	BCI Ear	Group Δ (BCI-SSD)	SE	t	*R*^2^	DF	*P*
All subjects (*N* = 55/group)							
1	Best matched	− 31.11	4.50	− 6.92	0.31	(1, 108)	< 0.001
2	Worse	− 28.55	4.45	− 6.41	0.28	(1, 108)	< 0.001
With data logging (*N* = 18/group)							
3	Best matched	− 31.18	5.95	− 5.24	0.45	(1, 34)	< 0.001
4	Worse	− 21.92	7.20	− 3.04	0.25	(1, 34)	0.005
With etiology (NSSD = 44/NBCI = 31)							
5	Best matched	− 40.44	4.84	− 8.35	0.49	(1, 73)	< 0.001
6	Worse	− 35.53	5.13	− 6.93	0.40	(1, 73)	< 0.001
With only AB or cochlear CIs (NSSD = 44/NBCI = 55)							
7	Best matched	− 31.19	4.85	− 6.44	0.30	(1, 97)	< 0.001
8	Worse	− 28.63	4.79	− 5.97	0.27	(1, 97)	< 0.001

### Moderators

Table 4 shows the moderations on the comparisons between SSD CI and BCI listeners.

**Table 4:** Moderations of group effects on SSD performance. Table presents model comparisons for each moderator from the main model. Group effects and *p* values are from the newer model output, where BCI listeners represented the intercept

Row	Moderator	BCI Ear	Group Δ (SSD-BCI)	*p* (group effect)	F_change_	*R*^2^_Δ_	DF	*p* (model comparison)
1	Age	Best matched	− 31.02	< 0.001	0.78	0.01	(3, 106)	0.472
2		Worse	− 28.64	< 0.001	1.59	0.02	(3, 106)	0.208
3	Time since implant	Best matched	− 34.47	< 0.001	1.67	0.02	(3, 106)	0.192
4		Worse	− 30.75	< 0.001	1.48	0.02	(3, 106)	0.233
5	Etiology	Best matched	− 50.71	< 0.001	1.60	0.04	(5, 69)	0.184
6		Worse	− 45.93	< 0.001	1.56	0.05	(5, 69)	0.195
7	Device usage	Best matched	− 8.54	0.700	0.85	0.01	(3, 32)	0.437
8		Worse	− 6.06	0.812	0.33	0.02	(3, 26)	0.720
9	Device manufacturer	Best matched	− 32.76	< 0.001	6.21	0.08	(3, 95)	0.003
10		Worse	− 31.07	< 0.001	3.18	0.05	(3, 95)	0.046

#### Age

The addition of age as a moderator on group did not significantly improve model fits, and the SSD listeners remained significantly worse than BCI listeners in CNC performance (Table 4, Rows 1 + 2). This suggests that the group effect cannot be explained by any differences in age.

#### Time Since Activation

SSD CI users exhibited significantly less time since activation in comparison to both the best matched ear of the BCI users, *t*(68.81) = 7.34, *p* < 0.001, 95% CI [5.78, 10.09], M_BCI_ = 14.86 years vs. M_SSD_ = 6.92 years, and to the date of implanting the lower performing ear *t*(67.16) = 5.15, *p* < 0.001, 95% CI [3.60, 8.15], M_BCI_ = 12.80 vs. M_SSD_ = 6.92. Although these are large absolute differences in years, it should be noted that this is well past a conservative estimate of a 12–18-month adaptation period. The moderation of time since activation did not significantly improve model fit (Table 4, Rows 3 + 4), and the effect of group was significant when time since activation was added to the model. This suggests that the CNC difference between SSD and BCI groups on CNC scores was not attributable alone to time since activation.

#### Etiology of Deafness

Although there appeared to be differences due to etiology, the inclusion of this moderation did not significantly improve model fits (Table 4, Rows 5 + 6), and the group effect remained strongly significant with etiology in the model. Thus, the group differences in CNC performance are not solely attributed to etiology.

#### Device Usage

Overall, in line with clinical observation, SSD listeners exhibited significantly less daily average CI use than BCI listeners across listening conditions (Best Match: *t*(32.91) = 3.95, *p* < 0.001, 95% CI [2.25, 7.04], M_BCI_ = 13.60 vs. M_SSD_ = 8.96; worse ear: *t*(25.35) = 2.58, *p* = 0.016, 95% CI [0.70, 6.25], M_BCI_ = 12.43 vs. M_SSD_ = 8.96). The addition of hours of CI use (and its moderation with group) did not significantly account for any new variance (***R***^**2**^_**Δ**_ ~ 0.01; Table 4, Rows 7 + 8). And there was no significant moderation of group by device use (Best Match: *b* = − 1.231, SE = 2.130, *p* = 0.568) or (Worse Match: *b* = − 1.610, SE = 1.774, *p* = 0.371). However, when device use was added to the model, the group effect was no longer significant (Table 4, Rows 7 + 8), but neither was the effect of device use (Best Match: *b* = 1.768, SE = 1.364, *p* = 0.204; Worse Match: *b* = 1.389, SE = 1.723, *p* = 0.428). This suggests that the effect of device usage on CNC performance is entirely shared with listener group. Consistent with the large differences in hourly CI usage between the groups, hourly device usage was a strong indicator of listener type.

#### Device Manufacturer

The addition of device manufacturer (Advanced Bionics vs. Cochlear) as a moderator on group significantly improved model fit for both matched models (see Table 3 for main models; Table 4, Lines 9–10). Specifically, in this improvement there was a main effect of manufacturer, with significantly worse overall CNC scores for users of Advanced Bionics devices (*B* = − 19.40, *SE* = 6.32, *t*(95) = − 3.07, *p* = 0.003). However, this same effect was not significant in the worse matched model (*B* = − 12.02, *SE* = 6.44, *t*(95) = − 1.87, *p* = 0.065). Additionally, there was no moderation of device manufacturer on the group effect for either model (Best Match: *B* = 5.25, *SE* = 10.35, *t*(95) = 0.51, *p* = 0.613; Worse Match: *B* = − 2.13, *SE* = 10.54, *t*(95) = − 0.20, *p* = 0.840). Importantly, the group effect was still significant with the inclusion of device manufacturer as a moderator (Table 4, Lines 9–10). An additional model examining only SSD CI listeners (where all three manufacturers were available) did not find a significant effect of manufacturer (including Med-El users) within the SSD group (F(2, 52) = 1.47, *p* = 0.238).

##### First vs Second Implant in Sequential BCI Listeners

**Fig. 2:**
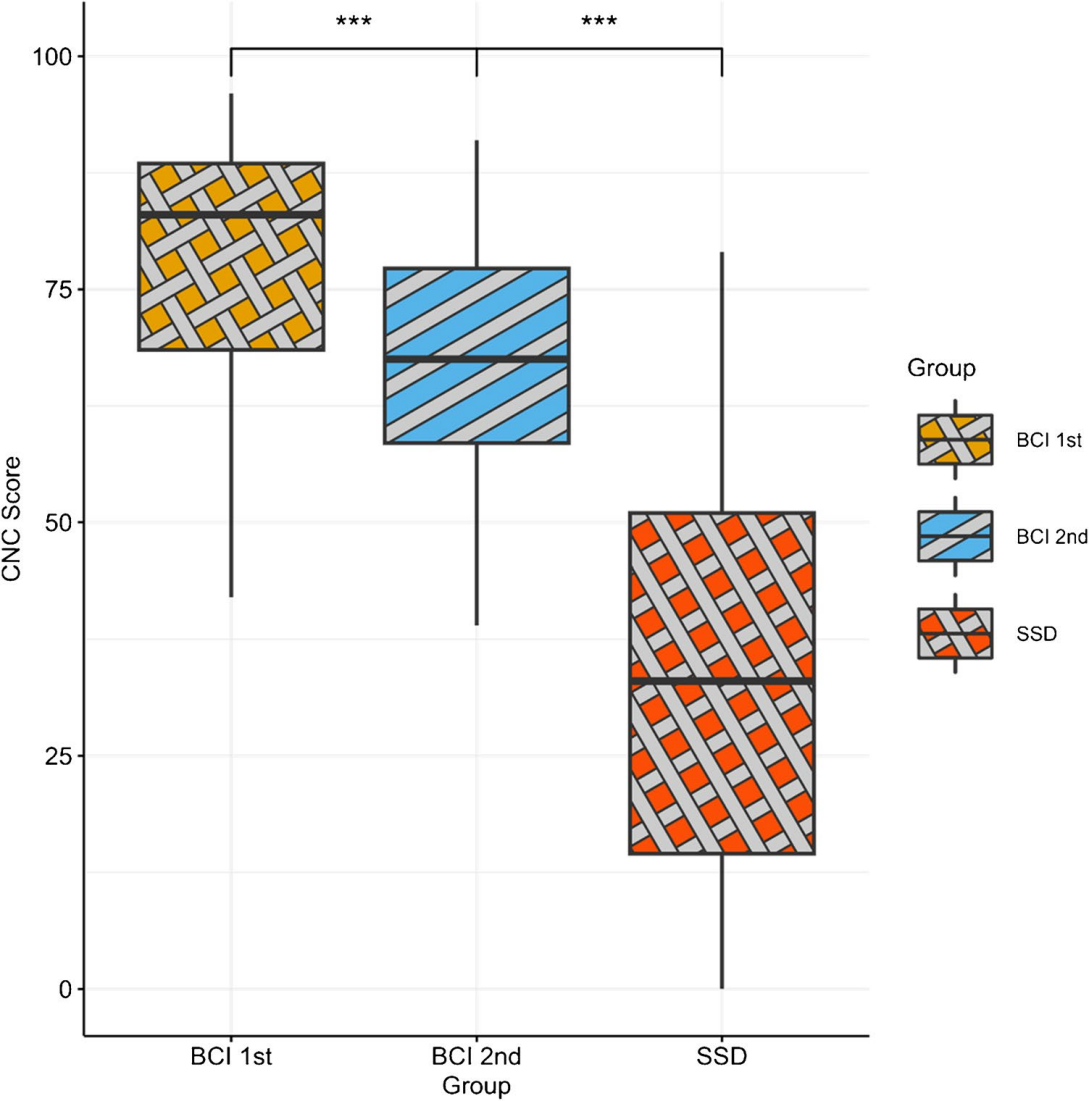
Comparison of CNC performance across listener groups (BCI sequential vs. SSD) and between implants in BCI sequential listeners

If the presence of a NH ear in SSD [partially] *blocks* the ability to adapt to the CI, this could also manifest *within BCI listeners*. That is, BCI listeners who were implanted sequentially might perform more similarly to SSD listeners on their second implanted ear (since its adaptation was blocked by the prior availability of the first implanted CI). This is a pattern that would be similar to the blocking of the adaptation of the CI by the prior availability of the NH ear in SSD listeners. To investigate this, a paired *t*-test compared the CNC performance between the second versus first ear within the 32 Sequential BCI listeners. A second between-subjects *t*-test compared the CNC performance between the second ear for sequential BCI listeners and that of SSD listeners to determine if the blocking effect within BCI users was larger than that shown by SSD CI listeners. Figure 2 displays the results. Sequential BCI listeners exhibited worse CNC scores in their second implanted ear than in their first ear, t(31) = 6.42, *p* < 0.001, M₁ = 75.57, M₂ = 64.13, Mdiff = 11.44, 95% CI [7.80, 15.07]. However, their CNC performance was still significantly higher compared to that of SSD listeners t(67) = − 5.98, *p* < 0.001, MBCI = 64.13, MSSD = 34.22, Mdiff = 29.91, 95% CI [− 39.88, − 19.93].

##### Asymmetric Bilateral Hearing Loss Compared to SSD Listeners

It is also quite possible that BCI listeners with asymmetric CNC performance across ears (hearing in one ear is substantially better than in the other) would rely on their better hearing ear in a similar way that SSD listeners rely on their NH ear. If this were the case, asymmetric BCI listeners should perform similarly to the SSD listeners on their worse performing ear. Therefore, an exploratory analysis focused only on the BCI listeners with the biggest difference between ears. This examined CNC performance from the worse performing ear for only BCI listeners for whom the disparity between ears was greater than > 11.42 (the mean disparity in these data). Age was also included as a moderator of group. This model found that SSD listeners still performed significantly worse than the asymmetric BCI listeners (SSD: b = − 15.92, SE = 5.77, t(73) = − 2.76, *p* = 0.00737). This suggests a lack of similarity between asymmetric BCI and SSD listeners. 

## Discussion

This study suggests that SSD CI listeners do not perform as well in speech perception with their CI alone as do age-matched BCI listeners listening with a single CI. This group difference persisted despite the inclusion of factors such as age, length of device use, etiology and device manufacturer in the models. Similar results have been seen for SSD CI listeners in prior work [39, 40, 47]. However, it is important to note that these studies did not directly compare SSD speech perception with that of BCI listeners. The control group in these studies consisted of CI users who were bilaterally deafened but who used only one CI. In this case, the fact that the SSD group — but not the control group — was listening with an atypical configuration (only one ear) could have heightened any differences between the groups. In contrast, our comparison controls for this and still shows a robust difference.

The number of hours recorded using the CI appeared to mediate group differences. Specifically, when this factor was added to the model, the group differences disappeared. However, adding device use to the model did not account for significant new variance over and above that of just group alone. Thus, this suggests that these effects share variance — SSD users do use their devices less and perform worse at speech perception. But the causal direction is not clear.

Device manufacturer significantly improved model fit across both matching conditions. However, this improvement was only driven by a significant main effect: Advanced Bionics users had overall worse CNC performance in the best matched condition. However, there were no other main effects or moderations of group across models. Additionally, there were no differences among manufacturers in the SSD group alone. Importantly, the group effect remained significant with the addition of this term in the models. These results suggests that the relationship between device manufacturer and CNC performance may be fairly inconsistent, mirroring prior research reporting mixed results with manufacturer comparisons [48]. Thus, we do not make strong claims about differences among CI manufacturers. Further research is needed to more comprehensively investigate the influence of manufacturer differences on CNC performance.

The underperformance of SSD CI listeners relative to BCI users suggests that SSD listeners may experience a *blocking* effect when adapting to their CI. That is, their normal hearing ear may be sufficient for speech perception in many daily tasks, creating less need to adapt the ear that perceives a degraded signal. Nonetheless, it should be noted that all these participants were tested at least 18 months post implantation, and many had quite a bit more (the average was over 6 years). Therefore, the strategies that SSD listeners are employing are not likely to be due to lack of experience with the implant. Supporting this, in this population of highly experienced users there were no significant overall effects of length of device use on CNC performance, suggesting that no further adaptation is likely to be seen.

This blocking effect could arise from several (not mutually exclusive) causes. It may be as simple as the fact that the NH ear perceives speech “better”, and the CI user simply turns off the CI in many situations. This is supported by their significantly lower daily usage rates. Alternatively, even if the CI is on, the NH ear may simply get more attention, again attenuating the experience. However, in addition to this, the introduction of a second “ear” with a highly distinct (and spectrally degraded signal), with a potentially misaligned place/pitch match [27, 49]. Additionally, the second ear could pose challenges to binaural integration [37], further leading listeners to ignore the CI ear. The CI ear could also create additional effort or fatigue. Such effects could lead a listener to use their CI less (further exacerbating the problem). That is, under either of this account, blocking is not inherent to the mechanisms of learning or adaptation, but arises from differential use (either real usage, or attentional differences). However, if this effect was only due to a matter of experience with the CI, 6 years at even a quarter of the daily usage (or attention levels) of a BCI listener would provide two years of functional experience—more than is needed for the adaptation period of a typical CI. Therefore, this concern should not significantly affect our results. Nonetheless, we tested this by asking if BCI users with more asymmetrical hearing show a profile more similar to SSD listeners. This prediction was not confirmed: CNC performance for BCI listeners with more asymmetry was still better than for SSD listeners. This supports our claim that any potential disparity between BCI listeners was not enough to account for the difference between groups.

Alternatively, blocking may be a consequence of error-driven learning [38]. In most learning and adaptation systems, learning is a function of the difference between the desired output of the system and its actual output. When this difference is low, little learning occurs. This is shown in classical conditioning experiments in which a learner first learns to pair one stimulus (e.g., a light) with a reward. When a second one is introduced (a tone is added to the light) they do not learn—even though it consistently predicts the reward. That is, when they later experience the second stimulus (the tone) alone, they show no evidence of learning. This was because the reward was already perfectly predictable from the first stimulus—there was no error signal present to drive learning. This form of error-driven learning—including the blocking effect—has been shown in learning novel speech categories [50], raising the possibility that may also apply here. SSD listeners offer a clear analog to this. In an SSD listener, the NH ear offers a clear and useful signal in most circumstances. As a result, even if the CI offers suboptimal input (that would induce high degrees of error in its own), the actual error signal is quite low, and little learning occurs.

Blocking may also be relevant in listeners with other forms of binaural integration. For example, in sequential BCI listeners, the first implanted CI is likely to have undergone some adaptation before the second CI is implanted. This adaptation is likely to be strong because there is no NH ear to fall back on: the error signal is high, and the adaptation has high stakes. However, when the second CI is added, performance is already good using the first CI, so there is less learning with the second CI. This is exactly what we observed in our exploratory analysis of the sequential BCI users in this study (Fig. 2). In contrast, simultaneously implanted BCI listeners must quickly adapt to the challenging auditory input provided by both CIs immediately following implantation; the error signal is high for both; they have no NH ear to fall back on. This may lead to a more robust adaptation. This hypothesis was supported by the fact that speech performance with the second implanted ear for sequential BCI listeners was significantly worse than that with the first implanted ear (Fig. 2), suggesting that BCI listeners could be relying on their better ear to perceive speech. However, it should be noted that this group of sequential listeners is too small to make strong claims.

These results have implications on audiological and clinical outcomes for SSD listeners who plan to receive a CI. Specifically, these results imply that SSD listeners may have unique needs for adaptation if they want to get the most out of their CI. Specifically, SSD listeners need more experience with their CI only to achieve equivalent levels of performance, and it may be important to control other strategies that they might use to minimize the error signal in these cases (e.g., subtitles) to promote better adaptation. Additionally, our results within sequentially implanted BCI listeners (that the first implanted ear performed better than the second ear), suggests there may be benefits of simultaneous implantation in this group (or at least simultaneous activation).

### Limitations

This study has several limitations worth noting. To start, analyses based on biological sex were not performed in this study since the comparison groups of interest (SSD vs. BCI) were generally matched on biological sex distribution. Due to this, there was little evidence for justification of biological sex influencing CI group differences in CNC scores.

Most importantly, we do not know what kind of auditory rehabilitation the listeners may have undergone. As part of the standard of care at our center, participants were recommended by their clinicians to do auditory training for 30 min a day for the first three months using the manufacturers rehabilitation programs. However, we did not have specific records of aural rehabilitation or auditory training in either group. Empirical and clinical work suggests a strong association between the persistent presence of auditory training and better speech perception outcomes [51–54].

Some experimental work suggests that adaptation can occur within a short period of time [55]. Since BCI listeners require more rehabilitation for both ears, it is quite possible that the BCI group had overall more access to training and rehabilitation, leading to the difference in speech perception between groups. The data logging analysis indexing CI usage in this paper helped to address part of this concern. This sample of data logging was small yet showed very large differences between groups. This makes it difficult to determine whether the effect of SSD listening was purely due to reduced experience/usage or derives from other causes. Additionally, without this specific information, it makes it difficult to determine the appropriate length of time (short or long) that SSD listeners would need to adapt to their implant. It is also possible that the differences observed in BCI participants with a large interaural difference may be related to differential device use between their two CIs. However, due to the limited availability of data logging, it is difficult to determine the extent to which device use may have contributed to these findings.

Additionally, all of the CI users had undergone CI processer tuning prior to speech perception testing, ensuring that they were in a similar state prior to testing. However, as we did not have access to aided audiometry for most participants, we cannot fully rule out the possibility that either the BCI and SSD groups were not using their processors at the right or consistent levels at test. Differences in processor volume or strategy between groups could have also contributed variance to the difference in speech perception.

Two factors prevented us from accounting for all etiological differences. First, for many patients in this sample, the etiologies were unknown. Second, there were many etiologies with only one or two patients — too few to make any definitive conclusions. However, we suspect that this is not a major factor, as in this specific study, the sample showed similar etiologies across the two groups despite expectations that there would be more single ear causes of deafness such as trauma or schwannoma. It may be important to evaluate CI only performance in listeners with a broader range of etiologies to determine if they play a role in adaptation. Moreover, with a highly diverse array of etiologies (and only a moderate sample), led to an analysis of etiology only by broad groups — a larger sample could potentially identify more specific factors that are relevant.

The focus of this study was entirely on single CI performance. However, the most important benefit of the CI is for binaural and spatial hearing. Thus, even as CI performance is lower than it could be, this may be outweighed by benefits of spatial hearing (or even of head shadow and squelch). Therefore, the results do not speak to whether there is overall benefit of SSD CI use. Rather the results are more focused on whether SSD CI users are achieving the same level of CI performance as other CI users.

Finally, the SSD listeners in this study achieved an average score of 34.9 on word recognition. It is important to note that this score is not 0, implying some degree of speech perception. These findings suggest that SSD listeners are indeed adapting to their CI, albeit through a potentially less efficient process when contrasted with their bilateral CI counterparts. Consequently, these results underscore the potential for growth and enhancement through intervention and training. However, a comprehensive understanding of the most effective auditory rehabilitation methods necessitates a deeper exploration of the fundamental mechanisms underlying binaural perception and its influence on speech perception in SSD CI listeners, and with binaural fusion.

## Conclusions

To conclude, SSD listeners adapt differently to their implant compared to BCI listeners, even after controlling for differences in device experience, etiology, and age. Specifically, they show a blocking effect on their adaptation where their reliance upon their NH ear to perceive speech in their daily lives prevents them from fully adapting to the CI. Further work should investigate whether or how binaural integration occurs in this population to help determine the trajectory of more thorough CI training supporting speech perception.

## Data Availability

The data and scripts for data analysis used in this study are available at the Open Science Framework listed at https://osf.io/vs6g3.

## Code Availability

The code used for data analysis and figure generation is provided in a script that has been made publicly available on the Open Science Framework at https://osf.io/vs6g3/.
